# Second primary malignancies after ocular adnexal lymphoma diagnosis

**DOI:** 10.1186/s12886-021-01921-7

**Published:** 2021-04-07

**Authors:** Asad Loya, Vignesh Ramachandran, Talha Ayaz, Christina Y. Weng

**Affiliations:** 1 Baylor College of Medicine, School of Medicine, 1 Baylor Plaza, Houston, TX 77030 USA; 2 University of Texas Medical Branch at Galveston, School of Medicine, 301 University Blvd., Galveston, TX 77555 USA; 3 Baylor College of Medicine, Department of Ophthalmology-Cullen Eye Institute, 1977 Butler Boulevard, Houston, TX 77030 USA

**Keywords:** OAL, ocular, adnexa, lymphoma, second, primary, malignancy, cancer, neoplasm, NHL

## Abstract

**Background:**

Although studies have investigated the risk of second primary malignancies (SPMs) associated with lymphoma of various sites, limited studies have investigated this risk in patients with lymphoma originating within the ocular adnexa. We conducted a retrospective study to assess incidence of secondary malignancies in patients with a prior diagnosis of ocular adnexal lymphoma (OAL) and to determine latency periods and age-groups at increased risk for SPM occurrence.

**Methods:**

Retrospective analysis was performed on data obtained from Surveillance, Epidemiology, and End Results (SEER) 9 database. Patients with an initial primary malignancy diagnosis of OAL between 1973 and 2015 were included in the study. Standardized incidence ratios (SIR) and excess absolute risks (EAR) compared to a SEER reference population with similar sex, race, age, and calendar year were computed for SPMs. Excess absolute risk is per 10,000 individuals; alpha of 0.05 was used.

**Results:**

Of 1834 patients with primary ocular adnexal lymphoma, 279 developed a secondary malignancy during average follow-up of 110.03 months (+/− 88.46), denoting higher incidence than expected (SIR 1.20; 95% CI, 1.07 to 1.35; EAR 30.56). Amongst the primary lymphoma cohort, 98.7% (1810/1834) of patients had non-Hodgkin’s lymphoma and amongst those that developed secondary malignancies, 99.6% (278/279) had non-Hodgkin’s lymphoma. Patients exhibited increased incidence of lymphohematopoietic and non-lymphohematopoietic second malignancies and no secondary malignancies of the eye or orbit. Patients had increased incidence of secondary malignancies in the first year (SIR 2.07; 95% CI, 1.49 to 2.79; EAR 150.37) and 1–5 years following lymphoma diagnosis (SIR 1.24; 95% CI, 1.01 to 1.51; EAR 34.89). Patients with various OAL subtypes demonstrated differing patterns of site-specific and overall SPM risk.

**Conclusions:**

Patients with prior diagnosis of ocular adnexal lymphoma possess increased risk of hematologic and non-hematologic secondary malignancies. Risk of secondary malignancy could vary by lymphoma subtype. Patients with ocular adnexal lymphoma may benefit from regular surveillance to promote early detection of second primary malignancies.

## Background

First described in 1952, ocular adnexal lymphoma (OAL), a subset of orbital lymphoproliferative disorders, is the most common malignancy involving the ocular adnexa and comprises 11–24% of all ocular tumors [1, 2]. OAL can originate from the orbit and surrounding structures, including the conjunctiva, orbital soft tissues, eyelid, lacrimal glands/drainage system or other adnexa [3]. These neoplasms are broadly classified as Hodgkin’s lymphoma (HL) and non-Hodgkin’s lymphoma (NHL), although NHL accounts for the vast majority of OAL. NHL is comprised of neoplasms derived from clonal proliferation of B or T lymphocytes of nodal or extranodal sites [4]. Most OALs are classified as low-grade B-cell NHL, and while most NHL arise from mature B lymphocytes (80%), T (14%), NK (6%), and plasma cell lines have also been implicated [5]. The most common major lymphoma subtypes responsible for OAL are extranodal marginal-zone B-cell lymphoma (EMZL) (55–66%), follicular lymphoma (FL) (10–29%), diffuse large B-cell lymphoma (DLBCL) (9–13%), mantle cell lymphoma (MCL) (6–11%), and small lymphocytic lymphoma (CLL/SLL) (2–5%) [6, 7].

Improvements in screening, early detection, palliative care, and definitive therapy have tripled the number of cancer survivors in the United States since 1971, and that number continues to grow at a rate of 2% annually [8]. Therefore, quantification of cancer sequelae and long-term effects of treatment, including second primary malignancy (SPM), has become increasingly important. OAL generally has favorable survival with a 5-year survival rate of 50–94%; however, many of these patients receive radiation, chemotherapy, or immunomodulation, which can increase susceptibility for SPMs [9, 10]. Patients with SPMs have worse survival compared to patients with isolated primary malignancies and may also suffer from sequelae such as vision loss and disfigurement [11, 12]. While the Surveillance, Epidemiology, and End Results (SEER) Program has been used to investigate SPMs in patients with other primary ophthalmic malignancies, [13, 14] little is understood about the risk of SPMs in patients with primary OAL.

In this study, the authors present a population-based study utilizing the SEER database to assess whether patients with primary OAL exhibit an increased incidence of SPMs compared to the general population. In doing so, the authors aim to assess types of SPMs that patients with history of OAL develop and to evaluate latency periods and age groups at particular risk for development of these SPMs.

## Methods

### Data extraction

Data was obtained from National Cancer Institute’s (NCI) SEER cancer registry database using Seer*stat (version 8.3.5) [15]. The database contains 18 registries, accounting for approximately 28% of the United States population. The dataset with 18 registries is limited to cases diagnosed after 2000, for which reason this study utilized the 9 registries dataset “SEER 9 Regs Research Data, Nov 2017 Sub (1973-2015),” containing cases diagnosed from 1973 to 2015. The database includes data related to patient demographics, tumor characteristics, treatment, and survival; it however lacks data related to modifiable risk factors (e.g., obesity, smoking), comorbidities, and specific treatment data (e.g., radiation dose, chemotherapy regimen). As only deidentified data was analyzed with permission from NCI, no institutional review board approval was required for this study in accordance with institutional policy.

### Data collection

All cases reporting a first primary tumor of OAL between 1973 and 2015 were included in the study. OAL was classified using the Collaborative Staging schema’s (v2.04) definition. Cases diagnosed at autopsy or by death certificate were excluded from the study. Only malignancies diagnosed 2 or more months after the first primary OAL diagnosis were considered as SPMs, in order to distinguish true secondary malignancies from multicentric lymphomas or concurrently diagnosed malignancies due to screening. Demographic variables collected included age group, race, and gender. Tumor characteristics collected include primary site, laterality, and histology. Vital status at end of follow-up period and surgical intervention were also documented.

### Statistical analysis

SPM analysis was conducted through a multiple primary standardized incidence ratio (MP-SIR) session using the Seer*stat program. Standardized incidence ratios (SIR) with 95% confidence intervals (95% CI) and excess absolute risks (EAR) compared to those in the general population with similar sex, race (white/unknown, black, other), age group (5-year interval), and calendar year (5-year interval) were computed for SPMs after OAL. Sub-analysis was performed using the same algorithm to compute SPMs after 5 specific OAL subtypes: EMZL, FL, DLBCL, MCL, CLL/SLL. The reference population used for the study was “SEER 9 1973-2015 (Nov 2017 sub), Race (WU/B/O), Event: Site recode B ICD-O-3/WHO 2008,” and is composed of Census Bureau data provided by SEER, in partnership with National Center for Health Statistics (https://seer.cancer.gov/popdata/). EAR was calculated per 10,000 individuals and a significance level of 0.05 was used. Descriptive analysis was conducted using IBM Statistical Package for the Social Sciences Statistics version 25.

## Results

### Cohort characteristics

Of 1834 patients with first primary OAL, 279 individuals developed a SPM during an average follow-up period of 110.03 months (+/− 88.46). Demographic, tumor, treatment, and survival information are displayed in Table 1. Orbit (not otherwise specified [NOS]) was the most common site of OAL within both the overall cohort (50.9%; 933/1834) and the SPM subset of the overall cohort (47.3%; 132/279). Majority of patients were managed without surgical intervention in the overall cohort (57.1%; 1047/1834), as well as in the SPM subcohort (58.4%; 163/279). Similarly, majority of patients received radiation therapy in both the overall cohort (64.3%; 1179/1834) and SPM subcohort (62.0%; 173/279). In contrast, chemotherapy was received by minority of patients in both the overall cohort (21.5%; 394/1834) and SPM subcohort (21.1%; 59/279). At the end of the follow-up period, 54.4% (998/1834) of patients from the overall cohort survived and 36.8% (101/279) of patients from the SPM subcohort survived.

**Table 1:** Demographic and tumor characteristics of patients at time of OAL diagnosis and survival status at end of follow-up period

		Overall Cohort		SPM Cohort	
		n	%	n	%
**Age Group**	0–49	373	20.3	30	10.8
	50–69	769	41.9	145	52
	70+	692	37.7	104	37.3
**Race**	White/Unknown	1504	82	233	83.5
	Black	143	7.8	17	6.1
	Other	187	10.2	29	10.4
**Gender**	Female	1015	55.3	141	50.5
	Male	819	44.7	138	49.5
**Primary site**	Conjunctiva	522	28.5	84	30.1
	Lacrimal Gland	220	12	37	13.3
	Orbit, NOS	933	50.9	132	47.3
	Eyelid	159	8.7	26	9.3
**Laterality**	Unilateral	1693	92.3	260	93.2
	Bilateral	119	6.5	15	5.4
	Unknown/NA	22	1.2	4	1.4
**Surgery status**	Performed	763	41.6	110	39.4
	Not Performed	1047	57.1	163	58.4
	Unknown	24	1.3	6	2.2
**Chemotherapy Status**	Received	394	21.5	59	21.1
	Not Received/Unknown	1440	78.5	220	78.9
**Radiation status**	Received	1179	64.3	173	62
	Not Received/ Unknown	655	35.7	106	38
**Vital status**	Living	998	54.4	101	36.2
	Deceased	836	45.6	178	63.8
**Type of cancer**	Hodgkin’s - Extranodal	2	0.1	0	0
	Plasmacytoma	22	1.2	1	0.4
	Non-Hodgkin’s - Extranodal	1810	98.7	278	99.6

*n* Sample size; *NA* Not applicable; *OAL* Ocular adnexal lymphoma; *NOS* Not otherwise specified; *SPM* Second primary malignancy

NHL was the predominant lymphoma sub-type amongst both the overall cohort (98.7%; 1810/1834) and SPM subcohort (99.6%; 278/279). The overall cohort was composed of 37.8% (693/1834) EMZL, 11.2% (206/1834) FL, 10.5% (192/1834) DLBCL, 10.0% (184/1834) CLL/SLL, and 3.8% (69/1834) MCL. The SPM subcohort was composed of 26.9% (75/279) EMZL, 14.7% (41/279) FL, 10% (28/279) DLBCL, 18.6% (52/279) CLL/SLL, and 4.3% (12/279) MCL. Majority of remainder cases in both the overall cohort and SPM subcohort were either not otherwise specified or of unknown lineage.

### SPM incidence

The incidence of malignancies was higher than that of the general population (SIR 1.20; 95% CI, 1.07 to 1.35; EAR 30.56). This can be attributed to increased occurrences of both hematologic and non-hematologic malignancies; SPMs of lymphohematopoietic origin included Hodgkin’s lymphoma (SIR 5.64; 95% CI, 1.16 to 16.47; EAR 1.6), non-Hodgkin’s lymphoma (SIR 5.29; 95% CI, 3.95 to 6.93; EAR 27.27), and leukemia (SIR 2.20; 95% CI, 1.23 to 3.62; EAR 5.28) whereas SPMs of non-lymphohematopoietic origin included non-epithelial skin sites (excluding melanoma and keratinocytic neoplasms; i.e. Merkel cell carcinoma, sebaceous adenocarcinoma, sweat gland adenocarcinoma) (SIR 4.91; 95% CI, 1.59 to 11.46; EAR 2.58), the kidney (SIR 2.08; 95% CI, 1.08 to 3.64; EAR 4.03), and the nervous system excluding brain (SIR 19.90; 95% CI, 2.41 to 71.89; EAR 1.23) (Table 2). Notably, there was a decreased incidence of female breast cancer (SIR 0.62; 95% CI, 0.37 to 0.97; EAR − 7.51). Additionally, no SPMs occurred in the eye or orbit (SIR 0; 95% CI, 0 to 10.64; EAR -0.22).

**Table 2:** Site-specific standardized incidence ratios and excess absolute risks of SPMs in patients with previous OAL

Second Cancer Site	O	O/E	95% CI	EAR**
All Sites	279	1.20*	1.07–1.35	30.56
All Sites excluding Non-Melanoma Skin	274	1.19*	1.05–1.34	27.98
All Solid Tumors	193	0.94	0.82–1.09	−7.46
Oral Cavity and Pharynx	2	0.39	0.05–1.42	−1.99
Digestive System	48	0.99	0.73–1.31	−0.3
Respiratory System	35	0.96	0.67–1.33	−1.01
Bones and Joints	1	4.8	0.12–26.73	0.51
Soft Tissue including Heart	1	0.84	0.02–4.68	− 0.12
Skin excluding Basal and Squamous	11	1.17	0.58–2.09	1.01
Melanoma of the Skin	6	0.71	0.26–1.55	−1.56
Other Non-Epithelial Skin	5	4.91*	1.59–11.46	2.58
Breast	19	0.62*	0.37–0.96	−7.68
Female Breast	19	0.62*	0.37–0.97	−13.29
Male Breast	0	0	0–14.22	−0.39
Female Genital System	12	0.99	0.51–1.73	− 0.16
Male Genital System	38	1.07	0.75–1.46	3.51
Urinary System	24	1.22	0.78–1.81	2.75
Urinary Bladder	12	0.94	0.48–1.64	−0.52
Kidney and Renal Pelvis	12	1.89	0.98–3.3	3.65
Renal Pelvis, Ureter, and Other Urinary Organs	0	0	0–3.12	−0.77
Kidney	12	2.08*	1.08–3.64	4.03
Renal Pelvis	0	0	0–6.24	−0.38
Ureter	0	0	0–9.7	−0.25
Other Urinary Organs	0	0	0–17.37	−0.14
Eye and Orbit	0	0	0–10.64	−0.22
Brain and Other Nervous System	2	0.87	0.11–3.15	−0.19
Brain	0	0	0–1.68	−1.42
Cranial Nerves/Other Nervous System	2	19.90*	2.41–71.89	1.23
Endocrine System	5	1.81	0.59–4.22	1.45
All Lymphatic and Hematopoietic Diseases	75	3.64*	2.86–4.56	35.17
Lymphoma	55	5.30*	4–6.9	28.87
Hodgkin’s	3	5.64*	1.16–16.47	1.6
HL - Nodal	3	5.86*	1.21–17.13	1.61
HL - Extranodal	0	0	0–180.57	−0.01
Non-Hodgkin’s	52	5.29*	3.95–6.93	27.27
NHL - Nodal	23	3.51*	2.22–5.26	10.63
NHL - Extranodal	29	8.84*	5.92–12.7	16.64
Myeloma	5	1.46	0.48–3.42	1.02
Leukemia	15	2.20*	1.23–3.62	5.28
Lymphocytic Leukemia	6	1.76	0.65–3.83	1.67
Acute Lymphocytic Leukemia	0	0	0–19.88	−0.12
Chronic Lymphocytic Leukemia	6	1.98	0.73–4.32	1.92
Other Lymphocytic Leukemia	0	0	0–18.31	−0.13
Non-Lymphocytic Leukemia	9	2.63*	1.2–5	3.61
Acute Non-Lymphocytic Leukemia (ANLL)	7	3.17*	1.28–6.54	3.1
Myeloid and Monocytic Leukemia	9	3.03*	1.39–5.75	3.9
Acute Myeloid Leukemia	6	3.17*	1.16–6.91	2.66
Acute Monocytic Leukemia	1	9.62	0.24–53.57	0.58
Chronic Myeloid Leukemia	1	1.14	0.03–6.38	0.08
Other Myeloid/Monocytic Leukemia	1	9.96	0.25–55.5	0.58
Other Leukemia	0	0	0–8.18	−0.29
Other Acute Leukemia	0	0	0–17.57	−0.14
Aleukemic, Subleukemic and NOS	0	0	0–15.29	−0.16
Mesothelioma	0	0	0–5.98	−0.4
Kaposi Sarcoma	0	0	0–19.49	−0.12
Miscellaneous	6	1.07	0.39–2.34	0.27

*CI* Confidence interval; *E* Expected; *HL* Hodgkin’s lymphoma; *NHL* Non-Hodgkin’s lymphoma; *O* Observed; *OAL* Ocular adnexal lymphoma; *SIR* Standardized incidence ratio; *SPM* Second primary malignancy. **Per 10,000 individuals. *P < 0.05

### SPM age and latency analysis

Patients with history of OAL demonstrated increased incidence of overall secondary malignancies within the first year (2–11 months) of OAL diagnosis (SIR 2.07; 95% CI, 1.49 to 2.79; EAR 150.37) and 1–5 years following OAL diagnosis (SIR 1.24; 95% CI, 1.01 to 1.51; EAR 34.89). Patients with age 0–49 years (SIR 2.41; 95% CI, 1.25 to 4.22; EAR 28.77) and 50–69 years (SIR 1.33; 95% CI, 1.08 to 1.62; EAR 38.98) had increased overall incidence of SPMs. In contrast, patients greater than 70 years (SIR 1.10; 95% CI, 0.94 to 1.28; EAR 23.15) demonstrated no difference.

Age groups with increased susceptibility to SPMs differed by malignancy origin (Table 3). Increased incidence of secondary NHL was found in the 0–49 years (SIR 8.34; 95% CI, 1.01 to 30.14; EAR 7.21), 50–69 years (SIR 8.44; 95% CI, 5.35 to 12.67; EAR 31.85), and greater than 70 years (SIR 3.93; 95% CI, 2.59 to 5.72; EAR 30.25) subgroups. On the other hand, increased incidence for SPMs of the kidney was found in the 0–49 years subgroup (SIR 12.77; 95% CI, 1.55 to 46.12; EAR 7.55), for non-epithelial skin in the 50–69 years subgroup (SIR 10.20; 95% CI, 1.23 to 36.83; EAR 2.83), for HL in the 50–69 years subgroup (SIR 10.93; 95% CI, 1.32 to 39.49; EAR 3.85), and for leukemia in the greater than 70 years subgroup (SIR 2.58; 95% CI, 1.38 to 4.42; EAR 11.98). Secondary malignancies of the nervous system (excluding brain) did not demonstrate a significant difference in incidence in any particular age group.

**Table 3:** Standardized incidence ratios and excess absolute risks of secondary malignancy distributed by time from diagnosis of the primary OAL

Cancer Site	Time Since Diagnosis							
	2–11 months		12–59 months		60–119 months		120+ months	
	O	O/E (95% CI)	O	O/E (95% CI)	O	O/E (95% CI)	O	O/E (95% CI)
All Sites	42	2.07* (1.49–2.79)	98	1.24* (1.01–1.51)	57	0.89 (0.68–1.16)	82	1.2 (0.95–1.48)
Other Non-Epithelial Skin	1	12.86 (0.33–71.66)	1	3.16 (0.08–17.6)	1	3.53 (0.09–19.69)	2	5.87 (0.71–21.2)
Female Breast	3	1.12 (0.23–3.27)	4	0.38* (0.1–0.98)	2	0.23* (0.03–0.83)	10	1.15 (0.55–2.11)
Kidney	4	8.31* (2.26–21.27)	4	2.1 (0.57–5.38)	3	1.89 (0.39–5.52)	1	0.56 (0.01–3.11)
Cranial Nerves/Other Nervous System	1	109.46* (2.77–609.86)	0	0 (0–103.57)	0	0 (0–131.8)	1	36.03 (0.91–200.74)
All Lymphatic and Hematopoietic Diseases	7	4.03* (1.62–8.31)	25	3.66* (2.37–5.41)	15	2.64* (1.47–4.35)	28	4.40* (2.92–6.36)
Lymphoma	3	3.45 (0.71–10.07)	19	5.53* (3.33–8.64)	10	3.48* (1.67–6.4)	23	7.21* (4.57–10.82)
Hodgkin’s Lymphoma	0	0 (0–75.01)	0	0 (0–19.72)	2	13.60* (1.65–49.11)	1	6.71 (0.17–37.39)
HL - Nodal	0	0 (0–77.88)	0	0 (0–20.49)	2	14.15* (1.71–51.13)	1	6.99 (0.18–38.92)
Non-Hodgkin’s Lymphoma	3	3.65 (0.75–10.68)	19	5.85* (3.52–9.14)	8	2.93* (1.27–5.78)	22	7.24* 4.53 10.95
NHL - Nodal	1	1.8 (0.05–10.02)	12	5.48* (2.83–9.58)	1	0.55 (0.01–3.06)	9	4.51* (2.06–8.57)
NHL - Extranodal	2	7.53 (0.91–27.22)	7	6.61* (2.66–13.63)	7	7.70* (3.09–15.86)	13	12.43* (6.62–21.26)
Leukemia	3	5.18* (1.07–15.14)	4	1.76 (0.48–4.52)	5	2.66 (0.86–6.2)	3	1.42 (0.29–4.16)
Non-Lymphocytic Leukemia	1	3.43 (0.09–19.08)	3	2.63 (0.54–7.68)	3	3.18 (0.66–9.31)	2	1.91 (0.23–6.91)
Acute Non-Lymphocytic Leukemia	1	5.39 (0.14–30.02)	3	4.13 (0.85–12.06)	2	3.29 (0.4–11.9)	1	1.46 (0.04–8.13)
Myeloid and Monocytic Leukemia	1	4 (0.1–22.28)	3	3.06 (0.63–8.94)	3	3.67 (0.76–10.71)	2	2.17 (0.26–7.85)
Acute Myeloid Leukemia	1	6.41 (0.16–35.74)	2	3.25 (0.39–11.76)	2	3.84 (0.46–13.86)	1	1.67 (0.04–9.3)

*CI* Confidence interval; *E* Expected; *HL* Hodgkin’s lymphoma; *NHL* Non-Hodgkin’s lymphoma; *O* Observed; *OAL* Ocular adnexal lymphoma; *SIR* Standardized incidence ratio; *SPM* Second primary malignancy. *P < 0.05

Similarly, latency periods with increased risk for secondary malignancies differed by origin (Table 4). Leukemia and SPMs originating in the kidney (SIR 8.31; 95% CI, 2.26 to 21.27) and nervous system (SIR 109.46; 95% CI, 2.77 to 609.86) demonstrated increased incidence within a year (2–11 months) of OAL diagnosis. NHL demonstrated increased incidence 1–5 years (SIR 5.85; 95% CI, 3.52 to 9.14), 5–10 years (SIR 2.93; 95% CI, 1.27 to 5.78), and 10+ years (SIR 7.24; 95% CI, 4.53 to 10.95) from OAL diagnosis. HL displayed increased incidence 5–10 years (SIR 13.60; 95% CI, 1.65 to 49.11) from OAL diagnosis. SPMs of the skin did not demonstrate any significant patterns regarding latency.

**Table 4:** Standardized incidence ratios and excess absolute risks of secondary malignancy distributed by age of patient

Cancer Site	Age at Diagnosis of Ocular Adnexal Lymphoma								
	0–49 years old			50–69 years old			70+ years old		
	O	O/E (95% CI)	EAR**	O	O/E (95% CI)	EAR**	O	O/E (95% CI)	EAR**
All Sites	12	2.41* (1.25–4.22)	28.77	99	1.33* (1.08–1.62)	38.98	168	1.1 (0.94–1.28)	23.15
Other Non-Epithelial Skin	0	0 (0–148.47)	−0.1	2	10.20* (1.23–36.83)	2.83	3	3.76 (0.78–11)	3.31
Female Breast	2	1.49 (0.18–5.37)	2.68	6	0.53 (0.19–1.15)	−8.45	11	0.61 (0.31–1.1)	−10.4
Kidney	2	12.77* (1.55–46.12)	7.55	5	2.32 (0.75–5.41)	4.47	5	1.45 (0.47–3.38)	2.33
Cranial Nerves/Other Nervous System	0	0 (0–453.34)	−0.03	1	26.25 (0.66–146.24)	1.51	1	18.43 (0.47–102.69)	1.42
All Lymphatic and Hematopoietic Diseases	3	6.24* (1.29–18.23)	10.31	29	5.24* (3.51–7.52)	36.86	43	2.95* (2.13–3.97)	42.69
Lymphoma	3	9.53* (1.97–27.86)	10.99	25	8.60* (5.56–12.69)	34.71	27	3.78* (2.49–5.5)	29.84
Hodgkin’s Lymphoma	1	13.34 (0.34–74.35)	3.79	2	10.93* (1.32–39.49)	3.85	0	0 (0–13.44)	−0.41
HL - Nodal	1	13.72 (0.35–76.42)	3.79	2	11.32* (1.37–40.89)	2.86	0	0 (0–14.06)	−0.39
Non-Hodgkin’s Lymphoma	2	8.34* (1.01–30.14)	7.21	23	8.44* (5.35–12.67)	31.85	27	3.93* (2.59–5.72)	30.25
NHL - Nodal	0	0 (0–23.93)	−0.63	8	4.33* (1.87–8.53)	9.66	15	3.29* (1.84–5.43)	15.7
NHL - Extranodal	2	23.37* (2.83–84.42)	7.84	15	17.12* (9.58–28.24)	22.19	12	5.18* (2.68–9.05)	14.55
Leukemia	0	0 (0–29.44)	−0.51	2	1.19 (0.14–4.31)	0.51	13	2.58* (1.38–4.42)	11.98
Non-Lymphocytic Leukemia	0	0 (0–48.13)	−0.31	1	1.31 (0.03–7.3)	0.37	8	3.10* (1.34–6.11)	8.14
Acute Non-Lymphocytic Leukemia	0	0 (0–79.16)	−0.19	1	1.99 (0.05–11.08)	0.78	6	3.62* (1.33–7.89)	6.53
Myeloid and Monocytic Leukemia	0	0 (0–51.61)	−0.29	1	1.44 (0.04–8.01)	0.48	8	3.63* (1.57–7.16)	−0.17
Acute Myeloid Leukemia	0	0 (0–89.14)	−0.17	1	2.25 (0.06–12.56)	8.71	5	3.56* (1.15–8.3)	0.87

*CI* Confidence interval; *EAR* Excess absolute risk; *E* Expected; *HL* Hodgkin’s lymphoma; *NHL* Non-Hodgkin’s lymphoma; *O* Observed; *OAL* Ocular adnexal lymphoma; *SIR* Standardized incidence ratio; *SPM* Second primary malignancy. **Per 10,000 individuals. *P < 0.05

### Sub-analysis by OAL subtype

SPM incidence further varied by OAL subtype (Table 5). Patients with initial FL (SIR 1.48; 95% CI, 1.06 to 2.01; EAR 79.48) and CLL/SLL (SIR 1.52; 95% CI, 1.13 to 1.99; EAR 84.31) were found to have increased incidence of malignancies relative to the general population, whereas patients with EZML (SIR 1.13; 95% CI, 0.89 to 1.41; EAR 17.23), DLBCL (SIR 1.38; 95% CI, 0.92 to 1.99; EAR 60.99), and MCL (SIR 1.20; 95% CI, 0.62 to 2.09; EAR 33.87) were found to have no difference in incidence of malignancies. Patients with prior FL or CLL/SLL demonstrated increased incidence of both hematologic and solid malignancies. Patients with prior EZML demonstrated increased incidence of hematologic but not solid malignancies, whereas those with prior DLBCL demonstrated increased incidence of solid but not hematologic malignancies. Conversely, patients with prior MCL were found to have no difference in incidence of either solid or hematologic malignancies relative to the general population.

**Table 5:** Site-specific standardized incidence ratios of SPMs in patients with varying prior OAL subtypes

	EZML		FL		CLL/SLL		DLBCL		MCL	
Second Cancer Site	O	SIR (95%CI)	O	O/E (95%CI)	O	O/E (95%CI)	O	O/E (95%CI)	O	O/E (95%CI)
All Sites	75	1.13 (0.89–1.41)	41	1.48* (1.06–2.01)	52	1.52* (1.13–1.99)	28	1.38 (0.92–1.99)	12	1.2 (0.62–2.09)
All Sites excluding Non-Melanoma Skin	74	1.12 (0.88–1.4)	40	1.45* (1.04–1.97)	52	1.52* (1.14–2)	28	1.38 (0.92–2)	11	1.1 (0.55–1.97)
Colon, Rectum and Anus	5	0.71 (0.23–1.65)	2	0.6 (0.07–2.15)	4	0.88 (0.24–2.25)	7	2.73* (1.1–5.63)	1	0.84 (0.02–4.68)
Respiratory System	6	0.58 (0.21–1.27)	5	1.12 (0.36–2.61)	11	2.02* (1.01–3.62)	0	0 (0–1.15)	3	1.85 (0.38–5.41)
Female Breast	5	0.54 (0.18–1.26)	3	0.81 (0.17–2.37)	0	0.00* (0–0.75)	5	1.73 (0.56–4.04)	1	1.02 (0.03–5.7)
Kidney	5	2.7 (0.88–6.3)	0	0 (0–5.41)	2	2.72 (0.33–9.81)	3	6.28* (1.3–18.37)	1	3.87 (0.1–21.55)
Cranial Nerves Other Nervous System	0	0 (0–125.28)	1	87.65* (2.22–488.37)	1	68.95* (1.75–384.18)	0	0 (0–418.03)	0	0 (0–857.12)
Thyroid	2	2.01 (0.24–7.25)	1	3.77 (0.1–21.02)	0	0 (0–12.59)	2	9.64* (1.17–34.84)	0	0 (0–43.31)
All Lymphatic and Hematopoietic Diseases	19	3.10* (1.87–4.85)	14	5.62* (3.07–9.43)	19	6.58* (3.96–10.27)	4	2.16 (0.59–5.53)	0	0 (0–4.17)
Non-Hodgkin Lymphoma	14	4.75* (2.59–7.96)	9	7.51* (3.43–14.25)	12	8.73* (4.51–15.25)	2	2.27 (0.27–8.2)	0	0 (0–9.03)
NHL - Nodal	9	4.69* (2.15–8.91)	3	3.73 (0.77–10.9)	2	2.13 (0.26–7.68)	2	3.4 (0.41–12.29)	0	0 (0–13.47)
NHL - Extranodal	5	4.84* (1.57–11.3)	6	15.21* (5.58–33.12)	10	23.03* (11.04–42.36)	0	0 (0–12.59)	0	0 (0–27.36)
Leukemia	5	2.54 (0.82–5.93)	3	3.59 (0.74–10.48)	5	5.14* (1.67–12)	1	1.59 (0.04–8.88)	0	0 (0–12.44)
Acute Non-Lymphocytic Leukemia (ANLL)	2	3.09 (0.37–11.17)	1	3.79 (0.1–21.1)	3	9.57* (1.97–27.98)	1	4.99 (0.13–27.79)	0	0 (0–38.77)
Acute Myeloid Leukemia	1	1.74 (0.04–9.68)	1	4.43 (0.11–24.67)	3	11.60* (2.39–33.91)	1	5.87 (0.15–32.72)	0	0 (0–45.66)
Acute Monocytic Leukemia	1	35.06 (0.89–195.36)	0	0 (0–296.17)	0	0 (0–237.19)	0	0 (0–386.43)	0	0 (0–803.64)
Chronic Myeloid Leukemia	1	4.01 (0.1–22.34)	0	0 (0–35.16)	0	0 (0–29.13)	0	0 (0–45.86)	0	0 (0–97.75)
Other Myeloid/Monocytic Leukemia	0	0 (0–158.88)	1	81.67* (2.07–455.04)	0	0 (0–223.64)	0	0 (0–372.52)	0	0 (0–810.25)

*CI* Confidence interval; *CLL/SLL* Chronic lymphocytic leukemia/small lymphocytic lymphoma; *DLBCL* Diffuse large B cell lymphoma; *E* Expected; *EMZL* Extranodal marginal-zone B cell lymphoma; *FL* Follicular lymphoma; *MCL* Mantle cell lymphoma; *O* Observed; *OAL* Ocular adnexal lymphoma; *SIR* Standardized incidence ratio; *SPM* Second primary malignancy. *P < 0.05

## Discussion

Using a population-based cancer database, we conducted a retrospective analysis of 1834 patients with OAL and found an increased incidence of overall SPMs compared to the general population, mostly within the first 5 years of OAL diagnosis and in individuals up to 70 years of age. Compared to the general population, patients with prior OAL diagnosis are at increased risk for lymphohematopoietic secondary malignancies such as HL, NHL, leukemia, and SPMs of solid sites such as in the kidney, non-epithelial skin (excluding melanoma and keratinocytic neoplasms), and nervous system (excluding brain).

While there is vast literature on occurrence of SPMs in patients with prior lymphoma, sparse data exists regarding SPMs in patients with prior lymphoma specific to the ocular adnexa. In this study, the majority (99.6%) of cases were characterized histologically as NHL. Chattopadhyay, et al. and Chien, et al. examined SPMs following NHL of non-specified sites and, similar to us, found an overall increased incidence of developing SPMs. Moreover, these authors found an increased incidence of SPMs at sites similar to those in our study, including kidney, skin, lymphoreticular system, and central nervous system [16, 17].

An increase in SPMs of the lymphoreticular system is likely attributable to shared pathophysiology between primary and secondary malignancies. Increased incidence of NHL was found after 1 year from primary OAL diagnosis. Specific OAL subtypes demonstrating this finding were EZML, FL, and CLL/SLL. As the majority of OAL cases in this study were NHL, this finding is more likely representative of local/regional/distant recurrence than true SPM occurrence, a distinction which is not well-defined in the SEER database; the database does not formally report recurrences, and a second primary tumor with the same histology would more likely represent a recurrence rather than a true SPM, especially in the case of lymphoproliferative disease. This finding is less likely due to synchronous or multicentric lymphoma, as increased incidence of NHL was found specifically following greater than 1-year latency periods. In contrast, HL demonstrated a unique latency period of 5–10 years between the time of primary OAL diagnosis and the SPM diagnosis, which may be due to potentially similar pathology and shared dysfunction of the lymphatic system; this finding was not specifically associated with any of the common OAL subtypes included in the sub-analysis, and may be associated with rarer OAL variants. Leukemia demonstrated increased incidence within a year from OAL diagnosis. This may be a result of increased cancer surveillance during the treatment and follow-up period immediately following cancer diagnosis leading to increased detection rather than a true increased incidence. On the contrary, studies [16, 17] examining NHL of non-specified sites also observed increased incidence of second primary leukemia, suggesting the validity of this finding which may be a result of shared pathophysiology or represent a transformation. Indeed, the most common OAL subtype associated with this finding was CLL/SLL, a tumor well-known for its proclivity to transform [18].

Shared risk factors, including genetic predisposition, or adverse long-term treatment effects may explain increased incidence of SPMs in certain sites [19]. The increased incidence of non-melanoma skin malignancies may be due to mutual risk factors such as UV exposure, [20, 21] whereas the increased incidence of SPMs of the kidney may be explained due to risk factors such as smoking, [22, 23] both of which are risk factors associated with NHL in general. SPMs of the skin did not demonstrate significant increase in incidence within any particular latency periods in this study. Conversely, SPMs of the kidney and nervous system (excluding the brain) exhibited significantly increased incidence within the first year following diagnosis of OAL. As with leukemia, this may result from detection bias rather than true increased incidence. Previous studies of NHL of non-specified sites did however report an increased incidence of secondary malignancies of these sites, signifying that these may indeed be true associations [16, 17].

We also demonstrated differences in SPM incidence based on patient age. It is important to note that SPMs of the kidney, non-epithelial skin, and HL were significantly increased in their respective age groups with an observed count of 2 malignancies each; clinical significance of these findings is indeterminate given the small numbers and that these malignancies could have arisen serendipitously. Other studies are necessary to validate these findings or increase their power through meta-analysis. While age-related cancer screenings do not presently exist for these malignancy types, the clinician should be aware of these potential associations.

Interestingly, there was a reduced incidence of breast cancer SPMs compared to the general population, with particular latency in the one to ten-year period following OAL diagnosis. Prior studies assessing sequence of diagnosis in patients with both breast cancer and NHL suggest that breast cancer is more frequently diagnosed simultaneously or prior to the diagnosis of NHL [24]. Because the objective of our study is to determine subsequent malignancies, our inclusion criteria is limited to only first primary OAL and therefore does not examine cases of breast cancer that predate an OAL diagnosis. Similarly, our analysis does not assess simultaneously diagnosed malignancies (within 0–2 months). It is therefore possible that this finding is reflective of such a unidirectional and sequential phenomenon rather than suggestive of an actual lower risk of breast cancer.

Additionally, there was no significant difference in incidence of non-lymphohematopoietic SPMs in the eye and orbit between patients with history of OAL and the reference population. This is consistent with what others have reported, and may be explained by inherent histopathological differences; lymphoma is distinct in histology from other types of ocular and periocular malignancies such as melanoma [16, 17]. Importantly, the absence of SPMs in this region suggests that treatment (e.g., radiation) is an uncommon cause of SPMs in patients with prior OAL.

Many second malignancy associations reported in studies examining NHL of any site were not observed in our study focusing solely on OAL. For example, Chattopadhyay, et al. have shown an increased risk for SPMs of the upper aerodigestive tract, gastrointestinal tract, lung, urinary bladder, melanoma, and thyroid gland [16]. Additionally, Chien and colleagues have found an increased incidence for SPMs of the stomach, head and neck, bone and soft tissue, thyroid, liver and biliary tract, and mediastinum [17]. The increased incidence of non-ocular mucosal malignancies in these studies may in part be explained due to our study including lymphomas solely of the ocular adnexa, whereas other studies included other primary sites of NHL such as the gastrointestinal tract. Extranodal lymphoma may originate from these non-ocular mucosal sites and require treatment, such as with radiation, which may predispose these patients to future increased risks of SPMs in these non-ocular mucosal sites. In comparison, OAL is remotely located and is less likely to possess treatment-related predisposition of SPMs in these regions. Alternatively, differences in our findings may be explained by differing distributions of NHL subtypes in the ocular adnexa compared to NHL in general; for example, whereas DLBCL constitutes 9–13% of OAL, it is the most common subtype of NHL in general (25–30%) [6, 7, 25]. Our sub-analysis demonstrated that patients with DLBCL of the ocular adnexa had increased incidence of colorectal and thyroid malignancies. Because DLBCL comprises a minor proportion of OAL, its relative effect may be masked by the other lymphoma subtypes that more commonly originate from the ocular adnexa and possess no relative difference in risk of colorectal or thyroid malignancies.

Although it has previously been documented that second primary malignancies following NHL do not differ by subtype, we found great variation of both overall and site-specific SPM risks between OAL subtypes in our sub-analysis [26]. Although this sub-analysis of SPM risk associated with specific OAL subtypes may prove helpful in explaining our overall findings, it must be interpreted with great caution, as sample sizes may represent as little as 3.8% (69/1834) of the overall cohort such as in the case of MCL. Future studies with larger sample size are necessary to reliably determine SPM risk associated with specific OAL subtypes.

The main limitations of this study, which utilizes the SEER cancer database, are its retrospective design and the inherent flaws associated with public databases. Our finding of overall increased risk of malignancies may be falsely elevated due to inclusion of likely misclassified NHL recurrences (18.6%; 52/279). The MP-SIR algorithm is unable to calculate SPMs without inclusion of all malignancies, limiting our ability to account for this confounding factor. Nonetheless, findings related to other site specific SPMs are highly reliable and of important clinical value. Additional issues include possible miscoding, underreporting (e.g., surgical treatment type), lack of variables related to radiation (e.g., dose, field, sessions), and unreliable or missing information regarding size and stage (e.g. 1834/1834 unknown Ann Arbor stage). Furthermore, limitations in reporting of chemotherapy (yes, no/unknown) and radiation (yes, no/unknown) data hinder our ability to explore treatment related effects. Risk of malignancy is affected by lifestyle-related factors such as smoking [22, 23, 27]. These factors are not recorded in SEER and would have strengthened our study if adjusted for. Additionally, patients may move from one region of the country to another and receive diagnosis and treatment for a SPM at a facility that does not report data to SEER. Therefore, our results may reflect overall underreporting in this regard, and there may be other types of SPM in patients with primary OAL that were not identified here. It is also possible that patients with less advanced disease are underrepresented, as ophthalmologists may be more comfortable treating these patients alone, without consultation from an oncologist or cancer center that collaborates with SEER; there is a lack of consensus, however, on how advanced initial disease impacts risk of SPMs, with early stage patients generally having longer survival periods in which they may develop SPMs and advanced stage patients receiving treatment that may elevates their risk for particular SPMs [28, 29]. Improved treatment options for lymphoma in the modern era have led to decreased SPMs that may affect outcomes in our study which encompasses cases diagnosed between 1973 and 2015 [30]. Despite these limitations, the SEER database has been validated for such analyses [31, 32].

## Conclusion

Our findings suggest that there is an increased risk of SPMs in patients with primary OAL, both of the lymphoreticular system and amongst solid sites. SPM risk may vary by OAL subtype. Patients with primary OAL may benefit from providers’ increased awareness of these risks. While additional investigation is needed, it may be prudent for healthcare providers (e.g., primary care, oncology, ophthalmology) of patients with prior OAL to monitor for high-risk SPMs during follow-up. Interdisciplinary coordination of care for each patient may facilitate early diagnosis and treatment, potentially improving long-term outcomes.

## Data Availability

The data that support the findings of this study are made available by National Cancer Institute’s Surveillance, Epidemiology, and End Results Program (SEER). Data can be obtained from SEER upon completing their agreement form and requesting access from their website (https://seer.cancer.gov). The specific dataset utilized was “SEER 9 Regs Research Data, Nov 2017 Sub (1973-2015).”

## Abbreviations

CI: Confidence interval
CLL/SLL: Chronic lymphocytic leukemia/small lymphocytic lymphoma
DLBCL: Diffuse large B-cell lymphoma
EAR: Excess absolute risk
E: Expected
EMZL: Extranodal marginal-zone B-cell lymphoma
FL: Follicular lymphoma
HL: Hodgkin’s lymphoma
MCL: Mantle cell lymphoma
NCI: National cancer institute
NHL: Non-Hodgkin’s lymphoma
NOS: Not otherwise specified
O: Observed
OAL: Ocular adnexal lymphoma
MP-SIR: Multiple primary standardized incidence ratio
SEER: Surveillance, epidemiology, and end results
SIR: Standardized incidence ratio
SPM: Second primary malignancy

## References

[1] Feinstein AR, Krause AC (1952). Ocular involvement in lymphomatous disease. AMA Arch Ophthalmol.

[2] Demirci H, Shields CL, Shields JA, Honavar SG, Mercado GJ, Tovilla JC (2002). Orbital tumors in the older adult population. Ophthalmology.

[3] Coupland SE, Krause L, Delecluse H-J, Anagnostopoulos I, Foss H-D, Hummel M (1998). Lymphoproliferative lesions of the ocular adnexa. Ophthalmology.

[4] Krol ADG, le Cessie S, Snijder S, Kluin-Nelemans JC, Kluin PM, Noordijk EM (2003). Primary extranodal non-Hodgkin’s lymphoma (NHL): the impact of alternative definitions tested in the comprehensive cancer centre west population-based NHL registry. Ann Oncol Off J Eur Soc Med Oncol.

[5] Swerdlow SH, Campo E, Harris NL, Jaffe ES, Pileri SA, Stein H. WHO classification of tumours of haematopoietic and lymphoid tissues, 4th ed. Lyon: IARC; 2008. p. 585. [cited 2018 Oct 29]. Available from: https://publications.iarc.fr/Book-And-Report-Series/Who-Classification-Of-Tumours/WHO-Classification-Of-Tumours-Of-Haematopoietic-And-Lymphoid-Tissues-2017.

[6] Holm F, Mikkelsen LH, Kamper P, Rasmussen PK, Larsen TS, Sjö LD, et al. Ocular adnexal lymphoma in Denmark: a nationwide study of 387 cases from 1980 to 2017. Br J Ophthalmol. 2020l ;bjophthalmol-2019-315637. [cited 2020 Nov 7] Available from: https://bjo.bmj.com/lookup/doi/10.1136/bjophthalmol-2019-315637 10.1136/bjophthalmol-2019-315637 32732342

[7] Ferry JA, Fung CY, Zukerberg L, Lucarelli MJ, Hasserjian RP, Preffer FI (2007). Lymphoma of the ocular adnexa: a study of 353 cases. Am J Surg Pathol.

[8] Travis LB (2006). The epidemiology of second primary cancers. Cancer Epidemiol Biomarkers Prev.

[9] Ng J, Shuryak I (2015). Minimizing second cancer risk following radiotherapy: current perspectives. Cancer Manag Res.

[10] Decaudin D, de Cremoux P, Vincent-Salomon A, Dendale R, Rouic LL-L (2006). Ocular adnexal lymphoma: a review of clinicopathologic features and treatment options. Blood.

[11] Vasudevan V, Cheung MC, Yang R, Zhuge Y, Fischer AC, Koniaris LG (2010). Pediatric solid tumors and second malignancies: characteristics and survival outcomes. J Surg Res.

[12] Abdulwahab A, Sykes J, Kamel-Reid S, Chang H, Brandwein JM. Therapy-related acute lymphoblastic leukemia is more frequent than previously recognized and has a poor prognosis. Cancer. 2012;118(16):3962–3967. [cited 2019 Mar 14] Available from: http://doi.wiley.com/10.1002/cncr.26735 10.1002/cncr.26735 22180297

[13] Tamboli D, Topham A, Singh N, Singh AD (2015). Retinoblastoma: a SEER dataset evaluation for treatment patterns, survival, and second malignant neoplasms. Am J Ophthalmol.

[14] Laíns I, Bartosch C, Mondim V, Healy B, Kim IK, Husain D (2016). Second primary neoplasms in patients with uveal melanoma: a SEER database analysis. Am J Ophthalmol.

[15] Surveillance, Epidemiology, and End Results (SEER) Program (www.seer.cancer.gov) SEER*Stat Database: Incidence - SEER 9 Regs Research Data (with additional treatment fields), Nov 2017 Sub (1973–2015) − Linked To County Attributes - Total U.S., 1969–2016 Counties, National Cancer Institute, DCCPS, Surveillance Research Program, released April 2018, based on the November 2017 submission.

[16] Chattopadhyay S, Sud A, Zheng G, Yu H, Sundquist K, Sundquist J, et al. Second primary cancers in non-Hodgkin lymphoma: Bidirectional analyses suggesting role for immune dysfunction. Int J Cancer. 2018;143(10):2449–2457. [cited 2019 Mar 13] Available from: http://doi.wiley.com/10.1002/ijc.31801 10.1002/ijc.31801 30238973

[17] Chien S-H, Liu C-J, Hong Y-C, Teng C-J, Hu Y-W, Ku F-C, et al. Development of second primary malignancy in patients with non-Hodgkin lymphoma: a nationwide population-based study. J Cancer Res Clin Oncol. 2015;141(11):1995–2004. [cited 2019 mar 13]. Available from: http://www.ncbi.nlm.nih.gov/pubmed/25971624. 10.1007/s00432-015-1979-1 PMC11824008 25971624

[18] Agbay RLMC, Jain N, Loghavi S, Medeiros LJ, Khoury JD. Histologic transformation of chronic lymphocytic leukemia/small lymphocytic lymphoma. Am J Hematol. 2016;91(10):1036–1043. [cited 2020 Nov 7] Available from: http://doi.wiley.com/10.1002/ajh.24473 10.1002/ajh.24473 27414262

[19] Brennan P, Scélo G, Hemminki K, Mellemkjaer L, Tracey E, Andersen A (2005). Second primary cancers among 109 000 cases of non-Hodgkin’s lymphoma. Br J Cancer.

[20] McMichael AJ, Giles GG (1996). Have increases in solar ultraviolet exposure contributed to the rise in incidence of non-Hodgkin’s lymphoma?. Br J Cancer.

[21] Cartwright R, McNally R, Staines A (1994). The increasing incidence of Non-Hodgkin’s Lymphoma (NHL): the possible role of sunlight. Leuk Lymphoma.

[22] Chow W-H, Dong LM, Devesa SS (2010). Epidemiology and risk factors for kidney cancer. Nat Rev Urol.

[23] Stagnaro E, Tumino R, Parodi S, Crosignani P, Fontana A, Masala G (2004). Non-Hodgkin’s lymphoma and type of tobacco smoke. Cancer Epidemiol Biomarkers Prev.

[24] Wiernik PH, Hu X, Ratech H, Fineberg S, Marino P, Schleider MA, et al. Non-Hodgkin’s lymphoma in women with breast cancer. Cancer J. 6(5):336–42 [cited 2019 Nov 13] Available from: http://www.ncbi.nlm.nih.gov/pubmed/11079174. 11079174

[25] Chihara D, Nastoupil LJ, Williams JN, Lee P, Koff JL, Flowers CR. New insights into the epidemiology of non-Hodgkin lymphoma and implications for therapy. Expert Rev Anticancer Ther. 2015;15(5):531–44. [cited 2020 Nov 7]. Available from: /pmc/articles/PMC4698971/?report=abstract. https://www.tandfonline.com/toc/iery20/current. 10.1586/14737140.2015.1023712 PMC4698971 25864967

[26] Rossi C, Jégu J, Mounier M, Dandoit M, Colonna M, Daubisse-Marliac L, et al. Risk assessment of second primary cancer according to histological subtype of non-Hodgkin lymphoma. Leuk Lymphoma. 2015;56(10):2876–2882. [cited 2019 Mar 13] Available from: http://www.tandfonline.com/doi/full/10.3109/10428194.2015.1007505 10.3109/10428194.2015.1007505 25641432

[27] Lutz JM, Cree IA, Foss AJ (1999). Risk factors for intraocular melanoma and occupational exposure. Br J Ophthalmol.

[28] Amer MH (2014). Multiple neoplasms, single primaries, and patient survival. Cancer Manag Res.

[29] Major A, Smith DE, Ghosh D, Rabinovitch R, Kamdar M (2020). Risk and subtypes of secondary primary malignancies in diffuse large B-cell lymphoma survivors change over time based on stage at diagnosis. Cancer.

[30] Lemieux MH, Solanki AA, Mahmood U, Chmura SJ, Koshy M. Risk of second malignancies in patients with early-stage classical Hodgkin’s lymphoma treated in a modern era. Cancer Med. 2015;(4):513–8 [cited 2019 Mar 13] Available from: https://www.ncbi.nlm.nih.gov/pmc/articles/PMC4402065/pdf/cam40004-0513.pdf. 10.1002/cam4.405 PMC4402065 25620577

[31] Singh AD, Turell ME, Topham AK (2011). Uveal melanoma: trends in incidence, treatment, and survival. Ophthalmology.

[32] Inskip PD. Multiple primary tumors involving cancer of the brain and central nervous system as the first or subsequent cancer. Cancer. 2003;98(3):562–570. Available from: http://doi.wiley.com/10.1002/cncr.11554 10.1002/cncr.11554 12879474

